# Stabilizers of edaravone aqueous solution and their action mechanisms. 2. Glutathione

**DOI:** 10.3164/jcbn.17-75

**Published:** 2017-10-26

**Authors:** Masahiko Tanaka, Satsuki Motomiya, Akio Fujisawa, Yorihiro Yamamoto

**Affiliations:** 1 School of Bioscience and Biotechnology, Tokyo University of Technology, 1404-1 Katakura-cho, Hachioji, Tokyo 192-0982, Japan

**Keywords:** edaravone, sodium bisulfite, cysteine, glutathione, deoxygenation

## Abstract

Edaravone (3-methyl-1-phenyl-2-pyrazolin-5-one) has garnered attention since its approval for amyotrophic lateral sclerosis in Japan (2015) and the United States (2017). Edaravone is administered intravenously, and as such, is distributed in the form of an aqueous solution. However, aqueous solutions of edaravone are very unstable because they present as edaravone anions, which become edaravone radicals when the anion donates an electron to free radicals including oxygen. In this study, glutathione (GSH) stabilized an aqueous edaravone solution during storage at 60°C for 4 weeks, and prevented the formation of potentially carcinogenic phenylhydrazine, while cysteine did not. One possible explanation is that GSH undergoes intermolecular hydrogen bonding with edaravone anions, while cysteine does not, as it favors intramolecular hydrogen boding. The combination of GSH and sodium bisulfite (NaHSO_3_) stabilized aqueous edaravone at room temperature for more than 1 year even under aerobic conditions. However, the U.S. Food and Drug Administration cautioned that NaHSO_3_ may cause allergic reactions. Therefore, we developed a stable edaravone aqueous solution without using NaHSO_3_, namely a combination of GSH with deoxygenation, which resulted in better stabilization of aqueous edaravone than the combination of GSH and NaHSO_3_.

## Introduction

**Fig. 1:**
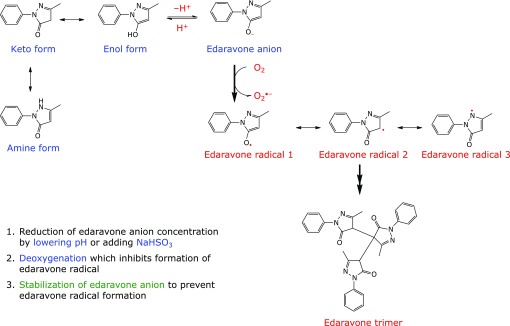
How to stabilize an aqueous solution of edaravone?

Edaravone (3-methyl-1-phenyl-2-pyrazolin-5-one) has recently garnered attention since it was approved for amyotrophic lateral sclerosis (ALS) in Japan (2015) and in the United States (2017).^(1,2)^ Edaravone was originally developed as a free radical scavenging drug, and was approved for the treatment of acute ischemic stroke in Japan in 2001.^(1,3)^ Because it is administered intravenously, it is distributed as an aqueous solution. Our previous study confirmed that the keto form of edaravone is the most stable form in non-polar solvent, but in water edaravone is present as the enol form and edaravone anion (Fig. 1).^(4)^ We also confirmed that aqueous edaravone is unstable because the edaravone anion becomes an edaravone radical when the anion donates an electron to oxygen (Fig. 1).^(4)^ The rate of this reaction is not fast but undoubtedly takes place. In fact, we observed the production of an edaravone trimer, which requires edaravone radicals as a precursor (Fig. 1).^(4)^

Obviously, the electron reduction of oxygen by the edaravone anion is the key step in edaravone degradation (Fig. 1). Thus, there are three ways to stabilize aqueous edaravone solution: reduction of edaravone anion concentration by lowering pH or adding sodium bisulfite (NaHSO_3_); deoxygenation, which inhibits formation of the edaravone radical; and stabilization of the edaravone anion to prevent edaravone radical formation.

Because we examined the first method in the previous paper,^(4)^ we discussed the last two methods in this study. In commercial products, aqueous edaravone solution is deoxygenated and kept at a pH ranging from 3.0 to 4.5 in the presence of NaHSO_3_ and cysteine.^(2,5)^ In this study, we observed that the addition of cysteine did not stabilize aqueous edaravone under aerobic conditions. On the other hand, the addition of glutathione (GSH) was effective despite the fact that both compounds are thiols. We also found that the combination of GSH with NaHSO_3_ was most effective for stabilizing edaravone even under aerobic conditions. However, the U.S. Food and Drug Administration (FDA) cautioned that NaHSO_3_ may cause allergic reactions,^(2)^ so we developed a stable edaravone aqueous solution without using NaHSO_3_.

## Materials and Methods

### Chemicals

Edaravone, cysteine, GSH, NaHSO_3_, phenylhydrazine (PHZ) hydrochloride, and other chemicals were of the highest grade commercially available. Water was purified with the Milli-Q Advantage system (Merck Millipore, Tokyo, Japan).

### Stability of edaravone in aqueous solutions

Edaravone (30 mg) was dissolved in 20 ml water. If necessary, 1 N aqueous sodium hydroxide was added, and the final pH was adjusted to 5–6 by adding 1 N aqueous HCl. The resulting 8.61 mM edaravone aqueous solution was mixed with either 20 mg NaHSO_3_ (9.61 mM), 10 mg cysteine (4.13 mM), 25.4 mg GSH (4.13 mM) or their combination. Aqueous edaravone solution was kept at 60°C or room temperature. An aqueous solution containing edaravone and GSH was purged with nitrogen (N_2_) gas and then stored in a tightly closed container.

### High-performance liquid chromatography analysis

Edaravone was quantified by high-performance liquid chromatography (HPLC) separation on the CAPCELL PAK ADME column (5 µm, 4.6 × 250 mm, Shiseido, Tokyo, Japan) using methanol/40 mM aqueous sodium phosphate (60/40 by volume) as the mobile phase (0.5 ml/min) with detection at 295 nm. PHZ was measured at the same HPLC conditions, with the exception of detection at 280 nm. GSH and its oxidized form (GSSG) were quantified by HPLC separation on the CAPCELL PAK C18 column (5 µm, 4.6 × 250 mm, Shiseido) using 3% methanol aqueous solution containing 0.05% trifluoroacetic acid as the mobile phase (1.0 ml/min) with detection at 210 nm.

### Statistical analysis

Data presented are mean values and standard deviations. Statistical analysis was performed with one-way ANOVA followed by the Scheffe’s multiple comparisons test. *p*<0.05 was considered statistically significant.

## Results and Discussion

**Fig. 2:**
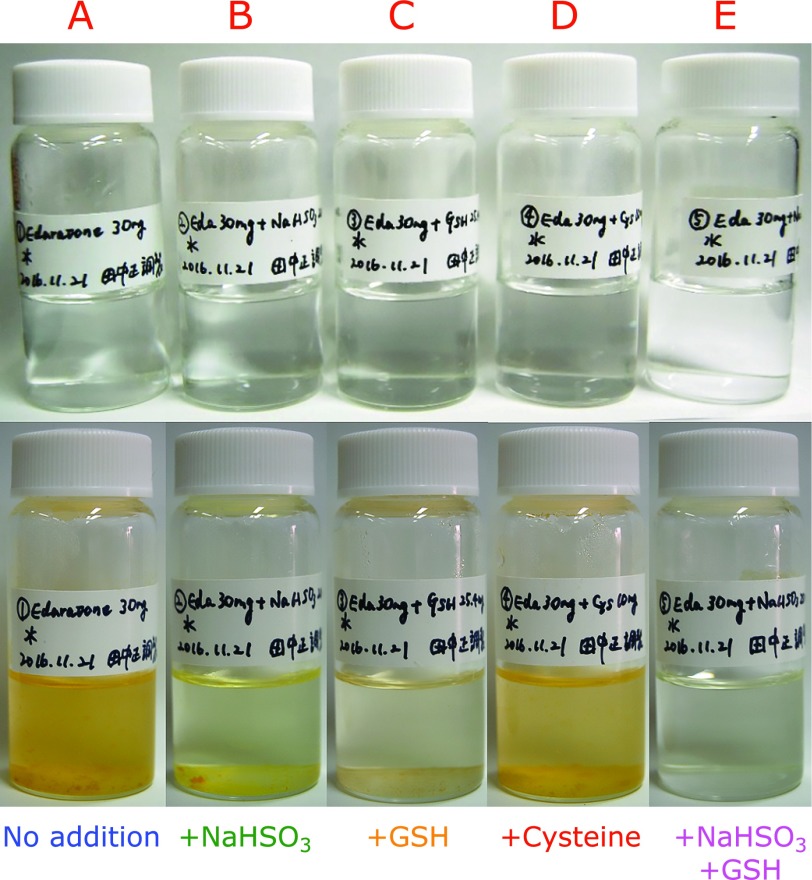
Precipitate formation during the storage of 8.61 mM edaravone in water at 60°C for 4 weeks under aerobic conditions (A) in the presence of 9.61 mM NaHSO_3_ (B); 4.13 mM GSH (C); 4.13 mM cysteine (C); 9.61 mM NaHSO_3_ and 4.13 mM GSH (D).

**Fig. 3:**
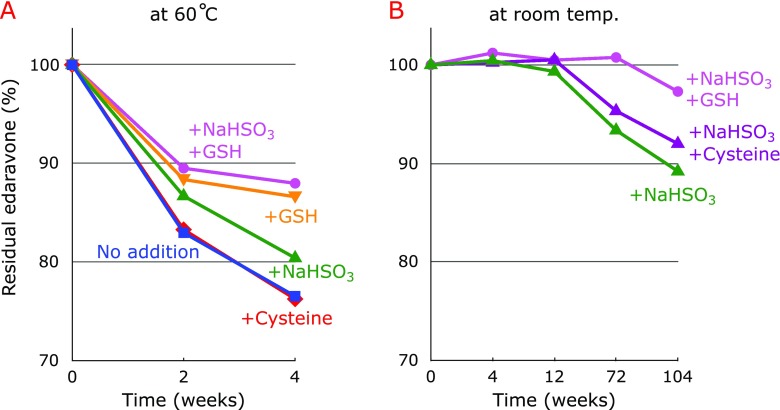
(A) Changes in edaravone concentration during the storage of 8.61 mM edaravone in water at 60°C for 4 weeks under aerobic conditions in the absence and the presence of 9.61 mM NaHSO_3_; 4.13 mM GSH and 4.13 mM cysteine; 9.61 mM NaHSO_3_ and 4.13 mM GSH. (B) Changes in edaravone concentration during the storage of 8.61 mM edaravone in water at room temperature for 104 weeks under aerobic conditions in the presence of 9.61 mM NaHSO_3_; 9.61 mM NaHSO_3_ and 4.13 mM cysteine; 9.61 mM NaHSO_3_ and 4.13 mM GSH.

Figure 2A shows precipitate formation during the storage of 8.61 mM edaravone in water at 60°C for 4 weeks. We previously found that this precipitate mostly consists of edaravone trimer and small amounts of edaravone.^(4)^ The addition of 9.61 mM NaHSO_3_ (Fig. 2B) and 4.13 mM GSH (Fig. 2C) partially prevented precipitate formation, whereas the addition of 4.13 mM cysteine had no effect (Fig. 2D). No significant precipitate was formed when 9.61 mM NaHSO_3_ and 4.13 mM GSH were added (Fig. 2E). Residual amounts of edaravone after 4 weeks of storage at 60°C decreased in the following order of additives used: NaHSO_3_ + GSH>GSH>NaHSO_3_>cysteine, as shown in Fig. 3A. This order correlated well with the amount of precipitate formation (Fig. 2). Surprisingly, cysteine did not stabilize aqueous edaravone, whereas GSH was effective. We will discuss the potential differences later. During storage at room temperature for 2 years under aerobic conditions, the residual amounts of edaravone decreased in the following order of additives used: NaHSO_3_ + GSH>NaHSO_3_ + cysteine>NaHSO_3_, as shown in Fig. 3B. These results confirm that GSH is better than cysteine as a stabilizer of aqueous edaravone. It is noteworthy that the combination of NaHSO_3_ and GSH inhibited the degradation of edaravone for more than 1 year even under aerobic conditions.

### PHZ formation

**Fig. 4:**
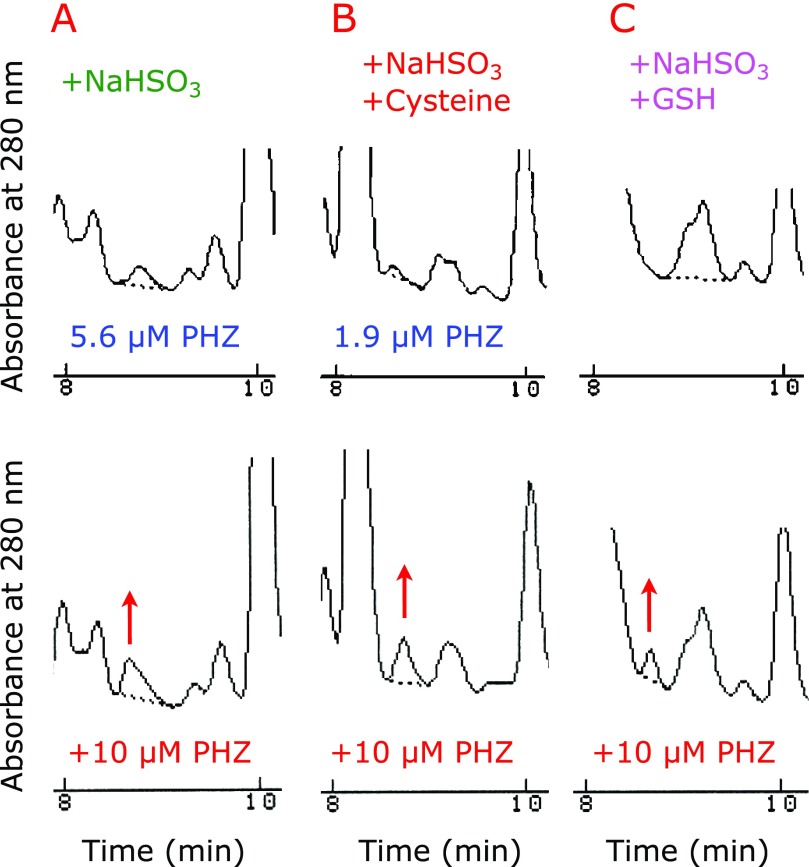
Formation of PHZ during the storage of 8.61 mM edaravone in water at 60°C for 5 weeks under aerobic conditions in the presence of 9.61 mM NaHSO_3_ (A); 9.61 mM NaHSO_3_ and 4.13 mM cysteine (B); 9.61 mM NaHSO_3_ and 4.13 mM GSH (C). Chromatograms show before (upper) and after the spiking of 10 µm PHZ (lower).

Edaravone is synthesized from PHZ and ethyl acetylacetonate.^(6)^ If edaravone is hydrolyzed, it yields potentially carcinogenic PHZ.^(7,8)^ Therefore, PHZ formation during storage should be prevented. Figure 4 shows the HPLC chromatograms of aqueous edaravone solutions after storage at 60°C for 5 weeks under aerobic conditions before and after spiking with 10 µM PHZ. It is clear that the addition of NaHSO_3_ (Fig. 4A) and NaHSO_3_ + cysteine (Fig. 4B) did not prevent PHZ formation. However, no formation of PHZ was observed when NaHSO_3_ + GSH were added to aqueous edaravone (Fig. 4C).

### The role of GSH in stabilizing aqueous edaravone solution

**Fig. 5:**
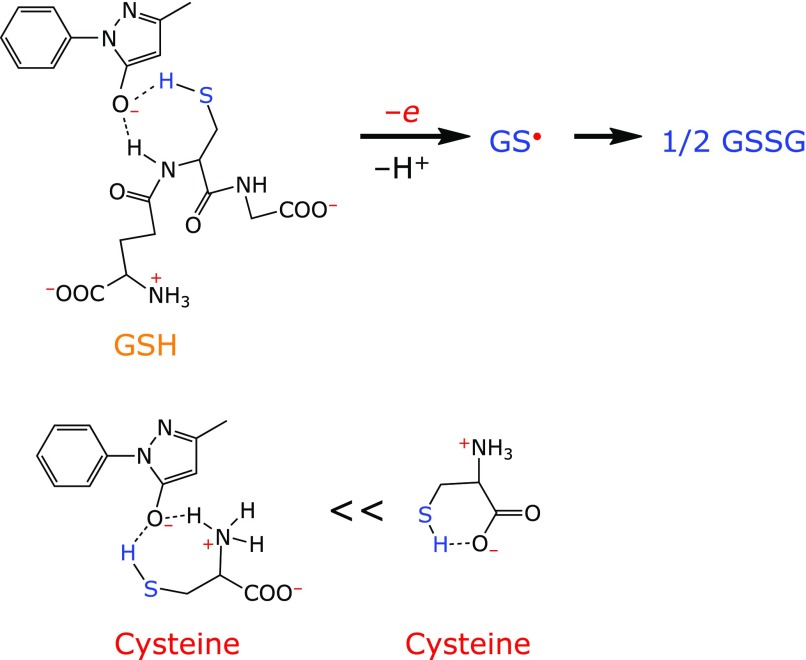
Intermolecular hydrogen bonding between edaravone anion and GSH and intramolecular hydrogen bonding in cysteine.

**Fig. 6:**
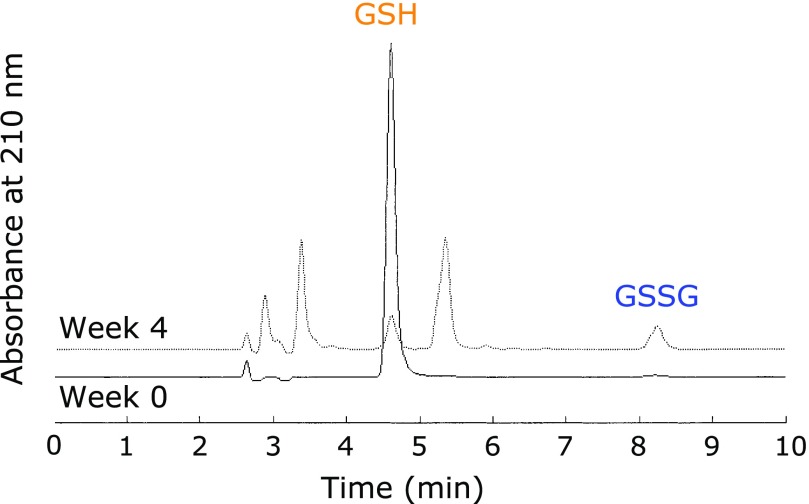
Decrease of GSH and GSSG formation during the storage of 8.61 mM edaravone in water at 60°C for 4 weeks under aerobic conditions in the presence of 4.13 mM GSH.

The above mentioned results demonstrate that GSH can stabilize edaravone anion, whereas cysteine does not. Therefore, GSH may donate an electron to oxygen instead of to the edaravone anion. This requires that GSH and the edaravone anion be located close to one another, probably by intermolecular hydrogen bonding as shown in Fig. 5. Donation of an electron from GSH yields a glutathione radical (GS^•^), and consequently, oxidized glutathione (GSSG). We confirmed the formation of GSSG after the storage of aqueous edaravone with GSH at 60°C for 4 weeks (Fig. 6). However, this notion should be studied more carefully since other degradation products of GSH were detected (Fig. 6). In the absence of edaravone, GSH was almost stable and no GSSG formation was observed after the storage 4.13 mM GSH at 60°C for 4 weeks (data not shown).

On the other hand, cysteine can form intramolecular hydrogen bonding as shown in Fig. 5, diminishing the probability of intermolecular hydrogen bonding with edaravone anion. However, it is difficult for GSH to engage in such intramolecular hydrogen bonding. Therefore, it appears that hydrogen bonding explains the difference between GSH and cysteine with regard to edaravone anion stabilization.

### NaHSO_3_-free stabilization of aqueous edaravone solution

**Fig. 7:**
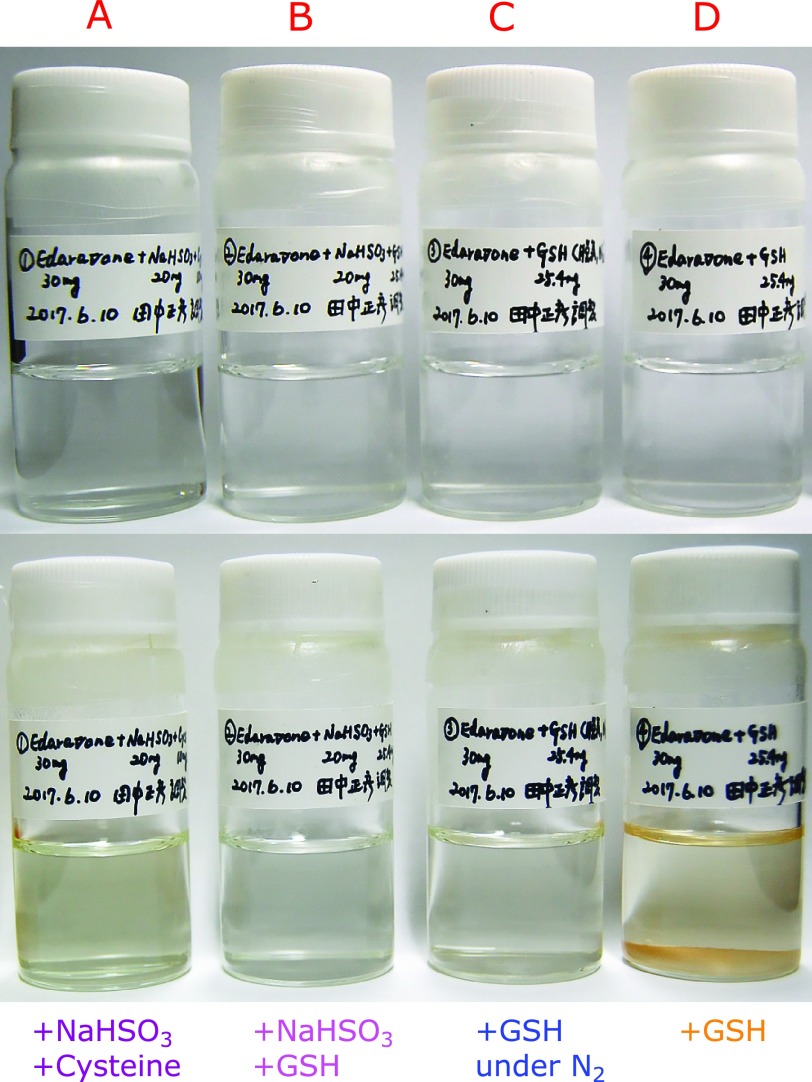
Precipitate formation during the storage of 8.61 mM edaravone in water at 60°C for 4 weeks under aerobic condition in the presence of 9.61 mM NaHSO_3_ and 4.13 mM cysteine (A); 9.61 mM NaHSO_3_ and 4.13 mM GSH (B); 4.13 mM GSH under N_2_ atmosphere (C); 4.13 mM GSH (D).

**Fig. 8:**
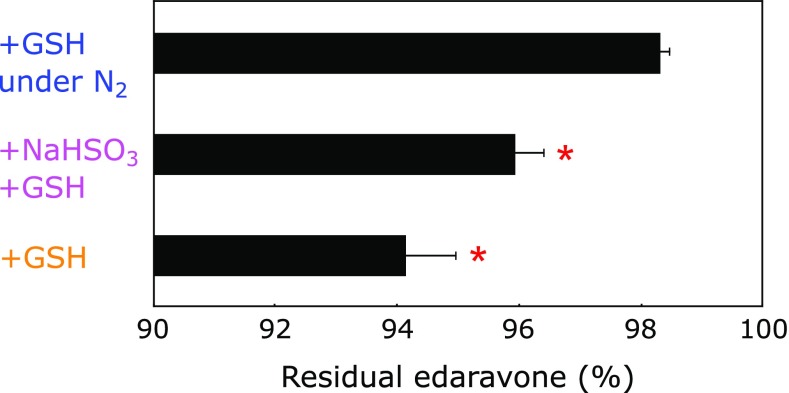
The percentage of residual edaravone after the storage of 8.61 mM edaravone in water at 60°C for 4 weeks under aerobic condition in the presence of 4.13 mM GSH; 9.61 mM sodium bisulfite and 4.13 mM GSH; 4.13 mM GSH under N_2_ atmosphere. * shows the significant difference (*p*<0.001) compared with the value obtained in the presence of GSH under N_2_ atmosphere, as analyzed by the Scheffe’s multiple comparison test. Bars and horizontal bars indicate mean + SD (*n* = 4).

Our results demonstrated that the combination of GSH and NaHSO_3_ was the most effective stabilizer of aqueous edaravone solution even in aerobic conditions. However, recent caution about using NaHSO_3_ put forth by FDA prompted us to establish a NaHSO_3_-free system. Because GSH was useful in stabilizing edaravone and preventing PHZ formation, we combined GSH with deoxygenation. Deoxygenation was achieved by purging oxygen with N_2_ gas. Precipitate formation was compared during the storage of aqueous 8.61 mM edaravone at 60°C for 4 weeks in the presence of 9.61 mM NaHSO_3_ and 4.13 mM cysteine (Fig. 7A), 9.61 mM NaHSO_3_ and 4.13 mM GSH (Fig. 7B) and 4.13 mM GSH (Figs. 7C and D). Samples were kept under aerobic conditions except 7C was under N_2_. It was clear that the combination of GSH and deoxygenation did not result in precipitation formation (Fig. 7C). Residual amounts of edaravone after 4 weeks of storage at 60°C decreased in the following order of additives used: GSH under N_2_>NaHSO_3_ + GSH>GSH, as shown in Fig. 8. Moreover, the combination of GSH with deoxygenation did not produce PHZ (data not shown).

Table 1 summarizes the efficacy of treatments for stabilizing aqueous edaravone solution. The addition of GSH was effective for the stabilization of aqueous edaravone, whereas cysteine was not effective. These differences are due to the intermolecular hydrogen bonding between GSH and the edaravone anion, whereas that type of interaction between cysteine and the edaravone anion is unlikely as cysteine favors intramolecular hydrogen bonding (Fig. 5). These differences may explain the fact that PHZ was inhibited by GSH addition but not by cysteine addition. The combination of GSH and NaHSO_3_ stabilized aqueous edaravone for more than 1 year at room temperature even under aerobic condition. However, we also demonstrated that the combination of GSH with deoxygenation, which satisfies the recent demand for NaHSO_3_-free stabilization of aqueous edaravone, was also effective for stabilization.

## Conclusions

GSH stabilized an aqueous edaravone solution during storage at 60°C for 4 weeks and prevented the formation of potentially carcinogenic PHZ, while cysteine did not. One possible explanation is that GSH undergoes intermolecular hydrogen bonding with the edaravone anion, whereas cysteine does not as it favors the intramolecular hydrogen boding. The combination of GSH and NaHSO_3_ stabilized aqueous edaravone at room temperature for more than 1 year even under aerobic condition. However, the combination of GSH with deoxygenation was also able to stabilize aqueous edaravone, and satisfies the recent demand for NaHSO_3_-free stabilization.

## Figures and Tables

**Table 1:** Efficacy of treatments on stabilization of aqueous edaravone

Additive	Efficacy
NaHSO_3_	△
Cysteine	×
NaHSO_3_ + cysteine	△
GSH	◯
NaHSO_3_ + GSH	◎
GSH + deoxygenation	◎

◎: good; ◯: moderate; △: marginal; ×: poor.

## Abbreviations

ALS: amyotrophic lateral sclerosis
FDA: the Food and Drug Administration
GSH: glutathione
GSSG: oxidized form of GSH
HPLC: high-performance liquid chromatography
PHZ: phenylhydrazine
